# Anti-idiotypic antibodies as cancer vaccines: achievements and future improvements

**DOI:** 10.3389/fonc.2012.00158

**Published:** 2012-11-06

**Authors:** Maha Z. Ladjemi

**Affiliations:** Institut de Recherche Expérimentale et Clinique, Pôle de Pneumologie, ORL and Dermatologie, Université Catholique de LouvainBrussels, Belgium

**Keywords:** anti-idiotype, antibodies, cancer, vaccines, solid tumors

## Abstract

Since the discovery of tumor-associated antigens (TAAs), researchers have tried to develop immune-based anti-cancer therapies. Thanks to their specificity, monoclonal antibodies (mAbs) offer the major advantage to induce fewer side effects than those caused by non-specific conventional treatments (e.g., chemotherapy, radiotherapy). Passive immunotherapy by means of mAbs or cytokines has proved efficacy in oncology and validated the use of immune-based agents as part of anti-cancer treatment options. The next step was to try to induce an active immune protection aiming to boost own’s host immune defense against TAAs. Cancer vaccines are thus developed to specifically induce active immune protection targeting only tumor cells while preserving normal tissues from a non-specific toxicity. But, as most of TAAs are self antigens, an immune tolerance against them exists representing a barrier to effective vaccination against these oncoproteins. One promising approach to break this immune tolerance consists in the use of anti-idiotypic (anti-Id) mAbs, so called Ab2, as antigen surrogates. This vaccination strategy allows also immunization against non-proteic antigens (such as carbohydrates). In some clinical studies, anti-Id cancer vaccines indeed induced efficient humoral and/or cellular immune responses associated with clinical benefit. This review article will focus on recent achievements of anti-Id mAbs use as cancer vaccines in solid tumors.

## INTRODUCTION

Since the discovery of tumor-associated antigens (TAAs), researchers have tried to develop immune-based anti-cancer therapies. Thanks to their specificity, monoclonal antibodies (mAbs) offer the major advantage to induce fewer side effects than those caused by non-specific conventional treatments (e.g., chemotherapy, radiotherapy). Passive immunotherapy by means of mAbs or cytokines has proved efficacy in oncology and validated the use of immune-based agents as part of anti-cancer treatment options (Ferrantini et al., 2007; Weiner et al., 2009). The next step was to try to induce an active immune protection aiming to boost own’s host immune defense against TAAs.

Active immunotherapy or vaccination is an antigen (Ag)-specific immune-stimulation. It consists in administering an Ag in the presence of an adjuvant. The role of the adjuvant is to induce a localized inflammatory reaction at the Ag administration site, making it more immunogenic. This adjuvant effect can be contained directly in the Ag, when it consists of an attenuated or dead infectious agent. Indeed, membranes substances (LPS) and DNA (CpG motifs) of infectious agents are able to activate the immune system. Currently, the artificial adjuvant most often used in vaccine preparations is aluminum hydroxide, while in mice the Freund’s adjuvant is most used.

In oncology, unlike non-specific active immunotherapy, the goal of vaccination is to stimulate a specific anti-tumor response targeting a TAA (Foon et al., 1999). The scientific rationale of this approach was that the immune effectors generated would be tumor-specific while preserving surrounding normal tissues from a non-specific toxicity.

The initial orientation of preclinical studies naturally targeted tumor-specific neoAgs; either strictly specific of the tumor (mutations, gene rearrangements, idiotypes), or Ags with expression restricted to tumor cells. However, as most of TAAs are self antigens, an immune tolerance against them exists representing a barrier to effective vaccination against these onco-proteins. One promising approach to break this immune tolerance consists in the use of anti-idiotypic (anti-Id) mAbs, so called Ab2, as Ag surrogates. The presence of anti-Id Abs acting as the internal image of antigen epitopes (Ab2 β) and the ability of anti-Id Abs to modulate the immune response have paved the way for many therapeutic processes in different areas such as autoimmune diseases or cancer research.

Anti-Id cancer vaccines are able to induce humoral and/or cellular immune responses. Indeed, clinical benefit was observed in patients enrolled in clinical trials testing anti-Id vaccines in oncology, particularly in patients who developed an immune response against the vaccine itself (Foon et al., 1998, 2000; Samonigg et al., 1999; Wagner et al., 2001).

This vaccination strategy requires very little equipment and allows vaccination against Ags from non-protein origin (such as carbohydrates) that are difficult to purify. Moreover, in preclinical models, the anti-Id Abs are particularly effective in breaking immune tolerance to certain TAA (Bhattacharya-Chatterjee et al., 2000; Saha and Chatterjee, 2010; Ladjemi et al., 2011). HAMA (human anti-mouse antibodies) type responses can be induced if the used anti-Id vaccine is from murine origin, but the techniques of Ab humanization (Losman et al., 1999) or the use of fully human Abs selected for example by phage display, can circumvent this problem.

In this review article, we will focus on recent achievements of anti-Id mAbs use as cancer vaccines in solid tumors. A first section will be dedicated to the concept of idiotypy and the anti-Id network theory first described by Jerne (1974). Indeed, according to this theory, the immune system is organized in Id and anti-Id network interactions able to regulate the immune response of the host against a given Ag. This particular feature of the immune system gave the idea to researchers to use the host’s immune system to break immune tolerance to oncofetal TAAs. One main advantage of anti-Id cancer vaccines among other vaccine strategy is their ability to target Ags from non-protein origin; a special focus will be given on recent achievements on anti-Id vaccines mimicking TAA from carbohydrate origin. We will then discuss on the anti-Id vaccines mimicking TAA from protein origin which are currently evaluated in clinical development.

## THE CONCEPT OF IDIOTYPY

### IDIOTYPY AND ANTI-IDIOTYPIC ANTIBODIES

The notion of idiotypy followed the experimental observations described simultaneously by Kunkel et al. (1963) in humans and by Oudin and Michel (1963) in rabbits. The idiotype is composed of a set of antigenic determinants or idiotopes (Poskitt et al., 1991b). The idiotope can be located on the variable light chain (Pasquali et al., 1987), the heavy chain (Parhami-Seren et al., 1990) or can result from the interaction of the two chains (Bentley et al., 1990). Five amino acids may be sufficient to define a linear idiotypic determinant (Attanasio et al., 1993). It was estimated that there were potentially 15–20 idiotopes per Ab molecule (Novotny et al., 1986), some of them being directly involved in the Ag binding site while others can be located outside the paratope (Jerne et al., 1982).

An idiotope is called private if it is expressed by a given specific Abs in an individual. Whereas in turn, idiotopes common to Abs produced by different individuals within a population or shared by Abs of different specificity in the same individual, define a public idiotope (recurring or cross-reactive idiotope; Poskitt et al., 1991a). The idiotopes represent one of the two identified epitope classes for mAbs, the other class being formed by the allotopes. Unlike allotopes, which are mostly located on the constant domains of light and heavy chains, idiotopes are found only in the hypervariable regions of Abs. In addition, idiotopes are from somatic origin unlike allotopes which are derived from the germline. The Ab2 anti-Id Abs are directed against idiotopes present on Abs or on receptors expressed on B lymphocytes; they can bind to idiotopes located at the site of Ag recognition.

### THE IDIOTYPIC NETWORK THEORY

**FIGURE 1:**
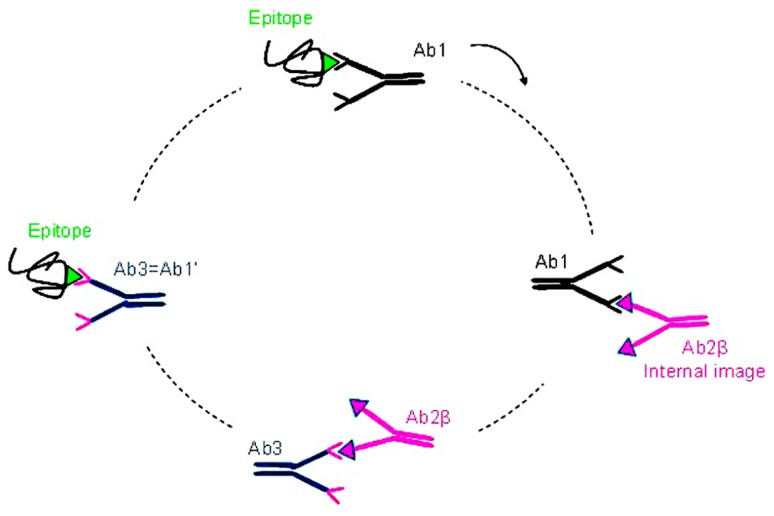
**The idiotypic network**. Immunization with a given Ag (green) generates the production of specific Abs directed against this Ag, theses first generation of Abs is named Ab1 (black). These Ab1 Abs can generate in turn the production of a series of anti-Id Abs directed against the Ab1 Abs and called Ab2. Some of these Ab2 Abs are able to mimic the three dimensional structure of the starting Ag, the Ab2 β (pink). Immunization with these Ab2 β Abs may lead to the production of anti-anti-Id Abs (Ab3 Abs) including the Ab1′ Abs (blue) directed against the corresponding initial Ag recognized by the Ab1.

Lindenmann (1973) and Jerne (1974) proposed theories describing the immune system as a network of interaction of Abs and lymphocytes. According to this hypothesis, Id and anti-Id network interactions would regulate the immune response of the host against a given Ag. The network theory is based on the fact that in the immune system the Ags are mimicked by idiotopes expressed by Abs and TCR (T cell receptors). According to this network concept (shown schematically in **Figure 1**) immunization with a given Ag will generate the production of Abs named Ab1 directed against this Ag. These Ab1 Abs can generate in turn the production of a series of anti-Id Abs directed against Ab1 Abs, so called Ab2. Some of these generated Ab2 Abs is able to mimic the three-dimensional structure of the starting Ag; the anti-Id Ab2 Abs constituting this subset are called Ab2 β. They are housed in the paratopes of Ab1 Abs and are able, when used as immunogens, to induce a specific immune response similar to that induced by the initial mimicked Ag.

Indeed, the β subtype of anti-Id Abs express the internal image of the Ag recognized by the Ab1 Ab and can therefore be used as Ag surrogates. Immunization with Ab2 β Abs may lead to the production of anti-anti-Id Abs, the Ab3 Abs, part of which recognizes the starting Ag recognized by the Ab1 Ab. Because of this Ab1-like reactivity, the Ab3 Abs are also called Ab1′ Abs to indicate that they may differ from the Ab1 Ab in their other idiotopes.

There are four subgroups of Ab2 Abs (α, β, γ, and ε) which can be obtained by immunization with Ab1 Abs:

– Ab2 α Abs are specific to idiotopes associated with the structure of the Ab and therefore outside the binding site, their binding to the Ab1 do not prevent the binding of Ag to the Ab1 (Jerne et al., 1982).
– Ab2 β Abs recognize an idiotope located in the paratope of the Ab1 Ab. Their binding to the Ab1 is naturally inhibited by the Ag they mimic. It is this series of Ab2 Abs that Jerne named “internal image” (Jerne, 1974) and Lindenmann the “homobodies” (Lindenmann, 1973). Antigenic determinants of the Ab2 β Abs are similar in structure to the Ag that binds the Ab1 Abs. These Ab2 β are able to induce the production of Ab3 Abs which could bind the original Ag.
– Ab2 γ Abs recognize idiotopes close to the paratope of the Ab1 Ab and their binding to the Ab1 is inhibited by the binding of Ag, mainly because of steric hindrance.
– Ab2 ε Abs or “epibodies” have double features as they can bind both the epitope of the Ag and the idiotope of the Ab directed against this Ag (the Ab1; Bona et al., 1986).

Of all Abs that can be produced against a given Ab1 Ab, the one representing the internal image of the Ag would constitute the best candidate to induce anti-tumoral immune response. In a preparation of polyclonal Ab2 Abs, only a subset of these Abs is able to mimic the Ag (the Ab2 β Abs). Thus, the induced fraction of Ab3/Ab1′ Abs will be much higher with monoclonal Ab2 β rather than with a polyclonal Ab2 Ab preparation (Herlyn et al., 1996). **Table 1** summarizes the clinical studies conducted with anti-Id mAbs as cancer vaccines in solid tumors.

**Table 1:** Clinical studies evaluating anti-idiotypic Abs as cancer vaccines in solid tumors.

Anti-Idiotype vaccine	Mimicked antigen	Tumor type	Clinical phase	Reference
Racotumomab (1E10)	NGc-containing	Breast cancer	I	Diaz et al. (2003), Guthmann et al. (2006)
	gangliosides	Lung cancer	l/l I	Neninger et al. (2007)
			Ongoing II/III	Alfonso et al. (2007), Hernandez et al. (2008)
		Melanoma?	I	Alfonso et al. (2002)
		Pediatric tumors?	Ongoing I	
TriGem	GD2	Melanoma	l/l I	Foon et al. (1995, 2000)
MK2-23	HMW-MAA	Melanoma	l/l I	Mittelman et al. (1994, 1995)
BR3E4	EpCam	Colorectal cancer	I	Birebent et al. (2001, 2003)
3H1 (CeaVac)	CEA	Colorectal cancer	l/l I III	Foon et al. (1995, 1997) Chong et al. (2006)
105AD7	CD55	Colorectal cancer	l/l I	Denton et al. (1994), Maxwell-Armstrong et al. (2001) Maxwell-Armstrong (2002)
11D10 (TriAb)	HMGF	Breast cancer	I	Reece et al. (2001, 2003)
		Colorectal cancer	II in association with 3H1	Posner et al. (2008)
		Lung cancer	II in association with 3H1	No data published yet
Abagovomab	CA-125	Ovarian cancer	l/l I	Reinartz et al. (2004), Pfisterer et al. (2006)
			Ongoing II/III	Sabbatini et al. (2006)

## ANTI-IDIOTYPIC VACCINES FOR GLYCOPEPTIDES CONTAINING TACA

A subset of TAA of carbohydrate nature have been identified and called tumor-associated carbohydrate antigens (TACA; Hakomori, 1989). TACA have been described to be expressed specifically on tumor cells as compared to normal tissues due to an aberrant glycosylation in tumor cells (Singhal and Hakomori, 1990). Many of TACA are expressed in fetal tissues and therefore belong to the group of the so-called oncofetal antigens. Moreover, TACA play an essential role for metastasis induction and tumor invasiveness. Number of TACA have been described, including the blood group related, mucin related TAA, the gobo series glycosphingolipids, and the gangliosides belonging to the group of sialic acid containing glycosphingolipids (Kobata and Amano, 2005; Guo and Wang, 2009; Cazet et al., 2010).

Gangliosides gained a privileged place as a target for cancer immunotherapy and recently in a study from the National Cancer Institute, 75 representative antigens to be targeted in cancer therapy were selected; among them four gangliosides GD2, GD3, fucosyl GM1, and *N*-acetyl-GM3 (Cheever et al., 2009). *N*-glycolyl (NGc) gangliosides received particular interest especially with the anti-Id murine mAb Racotumomab mimicking NGc-containing gangliosides (first known as 1E10 Ab).

### 1E10 Ab (RACOTUMOMAB)

Racotumomab, first known as 1E10 Ab, an anti-Id Ab2 γ murine mAb was generated after immunization of BALB/c mice with the P3 Ab1 IgM murine mAb (Perez et al., 2002; Lopez-Requena et al., 2003; Hernandez et al., 2005). Racotumomab have been used in several clinical trials and its safety and efficacy were assessed in different tumor localizations: melanoma, breast, and lung cancers. More recently, there was a specific interest on pediatric tumors expressing *N*-glycolylated gangliosides. A phase I study is indeed ongoing in patients with pediatric malignancies resistant to conventional treatment (NCT01598454) and the primary outcome will measure the higher safe dose level for ensuing clinical trials. Racotumomab has now reached the phase III clinical trials with possible indications in breast and lung cancer and a possible extent to pediatric tumors (Fernandez et al., 2010).

#### Racotumomab clinical evaluation in melanoma

A clinical trial with aluminum hydroxide-precipitated 1E10 Ab was conducted in 20 patients with advanced melanoma. 1E10 proved to be safe, well tolerated and able to induce specific immune responses against 1E10 itself and Neu-glycolyl-GM3 ganglioside (Alfonso et al., 2002).

#### Racotumomab clinical evaluation in breast cancer

The same formulation of 1E10-Alum used in melanoma was also evaluated a phase I clinical trial conducted in patients with stage III/IV breast cancer. Here again 1E10 Ab was safe, well tolerated and induced specific humoral immune responses both against 1E10 itself and NeuGc-GM3 ganglioside (Diaz et al., 2003). The toxicity and immunogenicity of 1E10 was investigated in a prolonged vaccination regimen in 19 patients with high-risk or metastatic breast cancer. 1E10 immunization induced a humoral response directed against NeuGc-GM3 ganglioside in all patients; moreover five patients developed specific T-cell responses (Guthmann et al., 2006).

#### Racotumomab clinical evaluation in lung cancer

A phase I clinical trial was conducted to evaluate the toxicity and humoral immune response elicited by aluminum hydroxide-precipitated 1E10 vaccine in nine patients with small cell lung cancer (SCLC). No evidence of serious adverse effects was found. Most of the patients developed specific antibody responses against both 1E10 Ab and NeuGc-GM3 ganglioside (Neninger et al., 2007). Although this study was not designed to evaluate the therapeutic efficacy of this anti-Id vaccine, a prolonged survival was observed in several patients. However, due to the small number of patients enrolled in this clinical trial, it did not allow to make a correlation between the induced immune response and clinical outcomes.

1E10/Alum formulation was also assessed in a clinical trial with stages IIIb and IV non-small cell lung cancer (NSCLC) patients. No evidence of unexpected or serious adverse effects was reported (Alfonso et al., 2007). Patients that developed IgG and/or IgM Abs against NeuGc-GM3 showed longer median survival times (Hernandez et al., 2008). Moreover, NeuGc-GM3-specific Abs were able to induce complement independent necrosis of tumor cells (Hernandez et al., 2011). A phase II randomized trial is now ongoing in NSCLC patients to confirm the clinical effect of 1E10 MAb vaccine and to evaluate the correlation between the immune responses patients’ survival (NCT01240447). A prospective randomized III study is also running in patients with advanced NSCLC, primary outcome will measure overall survival (OS; NCT01460472).

### TRIGEM Ab

Other clinical trials were performed with anti-Id mAbs mimicking gangliosides (Lutzky et al., 2002). Different doses of the anti-Id mAb TriGem mimicking disialoganglioside GD2, which is highly expressed in melanomas, were administered to patients. An initial study showed that of the 12 treated patients, one has a complete response and six showed an arrest of tumor progression (Foon et al., 1998). A second study conducted on 40 patients showed a complete response in one patient and stable disease in 12 of them (Foon et al., 2000). Furthermore, the data obtained suggest that this anti-Id mAb could have a favorable impact on disease progression and survival (Foon et al., 2000).

## ANTI-IDIOTYPIC VACCINES AS SURROGATES FOR SPECIFIC ONCOGENE PRODUCTS

We will detail in this section the use of Ab2 Abs mimicking specific TAA from protein origin as cancer vaccines in different types of solid tumors: melanoma, lung cancer, colorectal carcinoma, breast cancer, and ovarian carcinomas.

### MELANOMAS

The murine mAb MK2-23 mimicking the TAA HMW-MAA (high molecular weight-melanoma-associated antigen), was used to treat patients with advanced melanoma. The authors observed a regression of metastases (Mittelman et al., 1994) and an increase in overall survival correlated with the induction of an anti-HMW-MAA humoral response (Mittelman et al., 1995). Even though this was a retrospective study, a multivariate analysis showed that the development of anti-HMW-MAA antibodies was the most important variable for predicting survival. Wang et al. (2005) demonstrated that MK2-23 (mimic HMW-MAA)-IL-2 fusion protein is useful to implement active specific immunotherapy in patients with melanoma, because it bypasses the requirement for KLH conjugation and adjuvant administration.

Other clinical applications of various anti-Id Abs mimicking the HMW-MAA were conducted in small clinical trials. Among them, the Melimmune, a mixture of two murine mAbs, has proved effectiveness to induce Ag-specific humoral and cellular immune responses (Saleh et al., 1998). A more recent study has shown that this vaccine was able to induce specific cytotoxic T lymphocytes directed against the HMW-MAA (Murray et al., 2004).

### LUNG CANCER

As described above (see Idiotypy and Anti-Idiotypic Antibodies), the main anti-Id cancer vaccine tested in lung cancer remains Racotumomab with promising data. Two other anti-Id mAbs mimicking specific TAA from protein antigens are also evaluated in lung cancer patients: 3H1 (CeaVac) and 11D10 (TriAb) mAbs mimicking, respectively the carcinoembryonic antigen (CEA) and the human milk fat globule (HMGF) protein. A phase II study was conducted with 3H1 and 11D10 anti-Id mAbs in patients with completely resected Stage II and Stage IIIA NSCLC (NCT00006470). The objectives were the evaluation of toxicity, humoral and cellular immune responses, and to determine the progression-free survival (PFS) and OS in theses patients; results of this study are not published yet.

### COLORECTAL CANCER

During the past 20 years, studies on the use of anti-Id Abs have focused on three main tumor Ags (i) the epithelial cell adhesion molecule (EpCAM) associated with colorectal cancer (CRC), also known as GA733, CO17-1A, KS-14, or KSA, (ii) the CD55, also known as decay-accelerating factor (DAF) involved in the regulation of the complement cascade, and (iii) the CEA known for its particularly strong expression in 95% of CRC, 70% of lung adenocarcinomas, and 50% of breast cancers.

#### BR3E4

BR3E4, an Ab2 anti-Id mAb, was produced in rats against the murine mAb CO17-1A (Ab1; Herlyn et al., 1987; Maruyama et al., 2000). In a phase I clinical trial, BR3E4 was administered to 45 patients with CRC as intact IgG or as F(ab′)2 coupled to KLH (Birebent et al., 2001). This study demonstrated that there was a trend for the KLH group to induce higher immune response rates (18/21 and 5/15 patients with anti-anti-Id Abs and T cells, respectively) as compared to the group of patients immunized with the intact IgG (15/23 and 3/15 patients positive). However, clinical responses were rare as this study was undergone on stage IV CRC patients with liver metastasis (Birebent et al., 2001, 2003).

#### 3H1 (CeaVac)

An anti-Id murine Ab2 mAb, called 3H1 or CeaVac, mimicking an epitope of CEA was developed and very early suggested to be a potential cancer vaccine in patients with CEA-positive tumors (Bhattacharya-Chatterjee et al., 1990).

In a first clinical trial, the safety and immunogenicity of different doses (1, 2, or 4 mg) of aluminum hydroxide-precipitated anti-Id mAb were evaluated in 12 patients with advanced CRC (Foon et al., 1995). The vaccine was safe and well tolerated. Moreover, 3H1 was able to break immune tolerance to CEA in these patients with CEA-positive tumors (Foon et al., 1995). In fact, 9 of 12 patients developed anti-3H1 humoral responses and more interestingly all nine patients generated specific anti-CEA antibody responses. Moreover, 7 of 12 patients demonstrated idiotype-specific T cell proliferative responses and four also showed T cell proliferation to CEA (Foon et al., 1995). This 3H1 formulation was evaluated in a phase Ib trial in 24 patients with advanced CEA-positive CRC (Foon et al., 1997). The safety and immunogenicity of the vaccine confirmed the previous study. Even though the clinical benefit of 3H1 vaccine could not be proved in this study, since overall median survival (11.3 months) was comparable to other phase II data with advanced CRC patients treated with a variety of chemotherapy agents, including irinotecan, 3H1 vaccine promisingly induced considerably less toxicity (Foon et al., 1997).

3H1 anti-Id mAb is now evaluated in phase II and III clinical trials either alone or in combination with the anti-Id mAb 11D10 (TriAb) mimicking HMGF protein.

A phase III controlled randomized clinical trial was conducted on 630 patients with untreated metastatic CRC (Chong et al., 2006). The patients received 5-fluorouracil (5-FU) and leucovorin (LV) plus either 3H1 or placebo. The addition of 3H1 to 5-FU and LV did not result in increased toxicity but was not shown to improve overall patient outcomes (Chong et al., 2006). Anti-CEA antibody responses were observed in 70% of patients treated with 3H1; these patients had improved survival (median survival not reached) as compared to patients with a negative CEA response (median survival: 8.3 months; Chong et al., 2006).

The data of the first phase II clinical trial evaluating the combination of 3H1 and 11D10 anti-Id mAbs in patients with CRC metastatic to the liver were published (Posner et al., 2008). Vaccinations consisted of four biweekly treatments of 3H1 and 11D10, then monthly for 2 years, then on every other month for 3 years. The primary endpoint of the study was to investigate the proportion of patients recurrence-free at 2 years. The vaccine was well tolerated but did not improve 2-year recurrence-free survival when compared with the expected value of 40% reported for hepatic resection alone (Posner et al., 2008).

#### 105AD7

The cancer vaccine 105AD7 is a human anti-Id mAb that mimics the CD55 TAA on CRC cells. This anti-Id mAb was produced by fusion of a mouse/human heteromyeloma cell line with lymphocytes from a patient previously injected with mouse mAb 791T/36 for tumor immunoscintigraphy (Austin et al., 1989). Phase I studies in patients with advanced CRC demonstrated the safety and the immunogenicity of 105AD7 anti-Id mAb (reviewed by Maxwell-Armstrong, 2002). Immunization with 105AD7 induced T-cell immune responses in 83% of patients with a permissive haplotype (Durrant et al., 2000). In a phase I clinical trial, the cancer vaccine induced an increase in median-free survival for immunized patients versus unimmunized patients (12 vs. 4 months respectively; Denton et al., 1994). These results were not confirmed in a randomized double-blind phase II survival study (Maxwell-Armstrong et al., 2001). The reasons for lack of efficacy are unclear but with half of patients receiving only one or two doses of 105AD7, it seems to be insufficient to improve survival. A further survival analysis was then conducted but at 2-year follow-up, the vaccine did not improved OS (Maxwell-Armstrong, 2002).

### BREAST CANCER

Studies on the use of anti-Id Abs as cancer vaccines in breast cancer have focused on three main tumor Ags (i) the HMGF protein with 11D10 (TriAb), (ii) gangliosides with 1E10 (Racotumomab), and (iii) HER2 receptor (still in preclinical development).

The use of 11 D10 was evaluated in conjunction with autologous stem cell transplantation in patients with metastatic breast cancer (Reece et al., 2001, 2003). Immunization with 11D10 anti-Id mAb induced specific humoral and T-cell immune responses in the majority of patients (Reece et al., 2003). Moreover, patients with the most vigorous immune responses had a significant improvement in PFS (Reece et al., 2001)

The data on the use of anti-Id Abs mimicking HER2 as cancer vaccines for breast cancers are preliminary and still in the preclinical development.

Baral et al. (2001) have immunized C57BL/6 mice with the murine anti-Id mAb 520C9-6b mimicking a human epitope of HER2 Ag. The results of this preclinical study have shown that immunization with 520C9-6b could induce anti-HER2 Abs in vaccinated mice suggesting that this antibody could be used as a surrogate Ag of HER2 to induce a humoral and cellular response in patients with HER2-positive tumors. More recently, the same group developed and characterized a murine mAb 6D12, which mimics a specific epitope of HER2, the one recognized by trastuzumab. Immunization of C57BL/6 with 6D12 in combination with the adjuvant vaccine QS21, has led to the development of specific humoral responses. In addition, mice immunized with 6D12 were protected against a syngeneic graft of a lethal dose of the same cells (Mohanty et al., 2007; Pal et al., 2007). Moreover, immunization of transgenic mice tolerant to HER2 Ag with 6D12-pulsed dendritic cells (DC) could reverse Her-2/neu unresponsiveness and result in the induction of HER2/neu-specific humoral and cellular immune responses and protection against tumors expressing HER2/neu (Saha and Chatterjee, 2010).

Two human scFv fragments, named 40 and 69, and a llama anti-Id single domain antibody (sdAb), named VHH 1HE, capable of mimicking the epitope of HER2 Ag recognized by trastuzumab were selected by phage display. These anti-Id scFv and VHH fragments induced an anti-HER2 antibody response in BALB/c mice (Coelho et al., 2004; Alvarez-Rueda et al., 2009). Moreover, vaccination with anti-Id scFv fragments was able to reverse HER2 immunological tolerance and to protect HER2-tolerant mice from developing spontaneous mammary tumors (Ladjemi et al., 2011). Such vaccination elicited specific humoral and T-cell responses (Ladjemi et al., 2011).

### OVARIAN CANCERS

The use of the anti-Id mAb ACA-125 (or Abagovomab) mimicking the tumor antigen CA-125 (cancer Ag 125), over-expressed in ovarian tumors has been reported in several clinical trials. It has been shown, in preclinical and phase I clinical studies, that Abagovomab could induce humoral and cellular immune responses against CA-125 without the occurrence of toxicity related to this treatment (Wagner et al., 2001). In another phase Ib/II study, 119 patients with advanced ovarian cancer received 10 injections of Abagovomab; a CA125-specific humoral response was induced in 50% of patients. A positive correlation was also observed between the development of a specific humoral response and OS (Reinartz et al., 2004). It should be noted that preclinical data suggest that use of a fusion protein formed by the Abagovomab and IL-6 may induce a more robust CA125-specific humoral response (Reinartz et al., 2003). Other phase I clinical trials evaluated Abagovomab in recurrent ovarian cancer (Pfisterer et al., 2006) or primary peritoneal tumors (Sabbatini et al., 2006) and confirmed the encouraging results obtained with this anti-Id mAb. The efficacy of Abagovomab is currently evaluated in a phase III randomized, double-blind, placebo-controlled clinical trial for patients in complete remission after a stage III-IV CA-125 positive ovarian, fallopian tube or primary peritoneal cancer (Grisham et al., 2011; Pfisterer et al., 2011). The primary outcome will measure recurrence-free survival; safety, time course of immune response, and OS will be measured as secondary outcomes. Preliminary immunogenicity data for weeks 10 and 22 showed that 68 and 69% of patients were positive for Ab3 Abs (Grisham et al., 2011).

## CONCLUSION

Immunotherapy has nowadays an important place in oncology treatment. The major advantage of this type of strategy is the specific targeting of tumor Ags, which implies less toxicity and side effects compared to conventional therapies. Today, 12 therapeutic Abs were approved by FDA for the treatment of cancers. Nevertheless, the high number of patients treated by therapeutic Abs and the experience taken from the several clinical trials conducted in this field have pointed two major problems currently facing Ab-based immunotherapy: (i) the development of drug resistance by tumor cells and (ii) the need of repeated injections required to achieve a lasting therapeutic effect, which implies a high cost for this type of therapy. Cell-based immune therapy faces with technical difficulties as it is still hard to establish a cell-based immunotherapy because of the individualization of the procedure and technical conditions of relatively complex *ex vivo* culture. However, the technological progress that the sector is currently experiencing lets consider the cell-based immune therapy as a promising future therapeutic strategy.

Active immunotherapy or vaccination offers the main advantage of requiring fewer injections than for therapeutic Abs. More importantly, vaccines offer the establishment, theoretically, of a memory response that persists after the end of treatment and could prevent the occurrence of relapses. Nevertheless, this strategy is still in preclinical and clinical development. This delay, as compared to other immunotherapy strategies, could be explained at least in part by the fact that clinical trials currently conducted are not adequate with a vaccination strategy. Indeed, vaccines are tested in patients with advanced stages of disease with immune system already weakened by many cycles of chemotherapy already undergone. This implies that the clinical benefit of this type of therapeutic strategy is even more difficult to demonstrate. However, the increasing interest for anti-tumoral vaccination could accelerate the development of cancer vaccines and increase the number of vaccine candidates to be tested which implies a larger number of clinical trials and thus give rise ultimately to commercialization of vaccines cancer. In addition, with the recent approval of the first cancer vaccine sipuleucel-T by the FDA in 2010 for metastatic hormone-refractory prostate cancer, cancer vaccines are entering a new promising era. In fact, sipuleucel-T increased OS in a randomized phase III trial conducted in patients with advanced prostate cancer (Higano et al., 2009, 2010).

Overall, research on anti-Id cancer vaccines has greatly evolved over the past decades even though there is yet a lot to do in this field. This vaccination strategy requires very little equipment and allows vaccination against Ags from non-protein origin (such as carbohydrates). Anti-Id cancer vaccines present the advantage to address to the entire population (regardless of HLA) as compared to protein or peptide-based vaccines. Moreover, they are capable of inducing an immune response more robust, at least in theory, since it is formed of humoral but also cellular component. These advantages allow foreseeing a bright future for this type of vaccine strategy. However, although most of anti-Id cancer vaccines proved safety, tolerability, and immunogenicity, the clinical benefit remains to be proved. This proof of clinical benefit will be perhaps provided by the promising anti-Id mAbs Racotumomab and Abagovomab, which are now evaluated in phase III clinical trials.

## Conflict of Interest Statement

The author declares that the research was conducted in the absence of any commercial or financial relationships that could be construed as a potential conflict of interest.
